# Identification of lncRNA–miRNA–mRNA networks in circulating exosomes as potential biomarkers for systemic sclerosis

**DOI:** 10.3389/fmed.2023.1111812

**Published:** 2023-02-16

**Authors:** Xiaolin Sun, Tiantian Ding, Baoyue Wang, Zhifang Chang, Hongchang Fei, Lixia Geng, Yongfu Wang

**Affiliations:** The First Affiliated Hospital of Baotou Medical College, Inner Mongolia University of Science and Technology, Baotou, Inner Mongolia, China

**Keywords:** systemic sclerosis, circulating exosomes, lncRNA, bioinformatics analysis, biomarkers

## Abstract

**Objective:**

This study aimed to analyze potential biomarkers for systemic sclerosis (SSc) by constructing lncRNA–miRNA–mRNA networks in circulating exosomes (cirexos).

**Materials and methods:**

Differentially expressed mRNAs (DEmRNAs) and lncRNAs (DElncRNAs) in SSc cirexos were screened using high-throughput sequencing and detected with real-time quantitative PCR (RT-qPCR). Differentially expressed genes (DEGs) were analyzed using the DisGeNET, GeneCards, GSEA4.2.3, Gene Ontology (GO), and Kyoto Encyclopedia of Genes and Genomes (KEGG) databases. Receiver operating characteristic (ROC) curves, correlation analyses, and a double-luciferase reporter gene detection assay were used to analyze competing endogenous RNA (ceRNA) networks and clinical data.

**Results:**

In this study, 286 DEmRNAs and 192 DElncRNAs were screened, of which 18 DEGs were the same as the SSc-related genes. The main SSc-related pathways included extracellular matrix (ECM) receptor interaction, local adhesion, platelet activation, and IgA production by the intestinal immune network. A hub gene, *COL1A1*, was obtained by a protein–protein interaction (PPI) network. Four ceRNA networks were predicted through Cytoscape. The relative expression levels of *COL1A1*, ENST0000313807, and NON-HSAT194388.1 were significantly higher in SSc, while the relative expression levels of hsa-miR-29a-3p, hsa-miR-29b-3p, and hsa-miR-29c-3p were significantly lower in SSc (*P* < 0.05). The ROC curve showed that the ENST00000313807-hsa-miR-29a-3p-*COL1A1* network as a combined biomarker of SSc is more valuable than independent diagnosis, and that it is correlated with high-resolution CT (HRCT), Scl-70, C-reactive protein (CRP), Ro-52, IL-10, IgM, lymphocyte percentage, neutrophil percentage, albumin divided by globulin, urea, and RDW-SD (*P* < 0.05). Double-luciferase reporter gene detection showed that ENST00000313807 interacts with hsa-miR-29a-3p, which interacts with *COL1A1*.

**Conclusion:**

The ENST00000313807-hsa-miR-29a-3p-*COL1A1* network in plasma cirexos represents a potential combined biomarker for the clinical diagnosis and treatment of SSc.

## 1. Introduction

Systemic sclerosis (SSc) is an autoimmune disease that includes diffuse systemic sclerosis (dSSc) and localized systemic sclerosis (lSSc) (1). The clinical manifestations of SSc include skin, lung, gastrointestinal, and cardiovascular damage (1). Lung damage includes pulmonary fibrosis and pulmonary vascular disease, which are characterized by a non-expectorant cough and dyspnea (1). SSc is a rare disease that mainly occurs in women, with an annual incidence rate of 10–20 per million people and a prevalence rate of 30–300 per million people (1). Traditional therapy is a combination of immunosuppressants, such as mycophenolate mofetil or cyclophosphamide, while hematopoietic stem cell transplantation is also used to treat refractory SSc (2). In view of the limited treatment methods at present, the quality of life and prognosis of patients with SSc are poor, and the early diagnosis of SSc without skin lesions is difficult. Therefore, it is particularly important to find appropriate biomarkers to assist in diagnosis or use in targeted therapy.

At present, the pathogenesis of SSc is incompletely understood. In addition to possible genetic and environmental factors, three other major factors play a critical role in the pathogenesis of SSc: endothelial injury and fibroproliferative vasculopathy, immune system abnormalities, and fibroblast dysfunction (3). Wasson et al. (4) showed that overexpression of lncRNA HOTAIR in the dermal fibroblasts of SSc induced the expression of collagen and α-smooth muscle actin, activated the NOTCH pathway, and inhibited the expression of miRNA-34a. They also found that HOTAIR was upregulated in *in vitro* cultured myofibroblasts from patients with SSc and in skin biopsies from SSc patients, while miRNA-34a was downregulated in the dermal fibroblasts of SSc *in vitro*. Mounting evidence has suggested that exosomes (EXOs) are closely related to vascular injury, immune abnormalities, and fibrosis in SSc (3). Circulating exosomes (circexos) are membrane-bound vesicles with a diameter of 30–120 nm that are secreted from tissue cells into blood (5). Cirexos can fuse with the cell membrane and transport proteins, lipids, and nucleic acids of the recipient cell for communication between cells or between cells and the environment, thus affecting the physiological and pathological functions of both the recipient cell and parent cell (6, 7). Thus, cirexos may be a novel and disease-specific biomarker for the diagnosis of SSc (8). Several miRNAs associated with profibrosis have been found to be significantly increased in cirexos isolated from the serum of patients with SSc, and serum cirexos isolated from patients with SSc stimulated the expression of genes encoding extracellular matrix components, such as collagen type I alpha 1 (*COL1A1*), collagen type III alpha 1 (*COL3A1*), and fibronectin 1 (9). Serum exosomes from patients with dSSc and lSSc also induced dose-dependent increases in the expression of genes related to myofibroblast activation (9). Therefore, cirexos may be involved in fibrosis in SSc.

Recently, researchers have paid increasing attention to competing endogenous RNAs (ceRNAs). ceRNAs do not specifically refer to one type of RNA, but rather a novel mechanism for RNAs interacting with each other. ceRNAs are involved in various diseases, such as cancer, immune-related diseases, cardiovascular diseases, and neurological diseases (10). Wang et al. (11) identified the regulatory role of lncRNA SNHG16 in myasthenia gravis (MG), an autoimmune disease, by constructing the ceRNA network. They found that SNHG16 was upregulated in patients with MG and was involved in its pathogenesis by competitively binding let-7c-5p to increase the expression of IL-10. Based on ceRNA, Fan et al. (12) found that lncRNA LOC100912373 could upregulate the expression of pyruvate dehydrogenase kinase 1 (*PDK1*), accelerate the phosphorylation of protein kinase B (AKT), induce the proliferation of fibroblast-like synoviocytes by competitively binding to miR-17-5p, and promote the occurrence and development of rheumatoid arthritis (RA). Extracellular lncRNAs are mainly enriched in exosomes, and cirexos can act as a protective barrier shielding lncRNAs from extracellular degradation, thus making lncRNAs stably expressed and easily detected as a biomarker of disease (13). LncRNAs, miRNAs, and mRNAs carried by cirexos have the potential to become new biomarkers and therapeutic targets of SSc (8, 14). However, there are limited studies on lncRNAs in cirexos in SSc and other autoimmune diseases, and many lncRNAs in plasma cirexos have not been fully studied.

**FIGURE 1:**
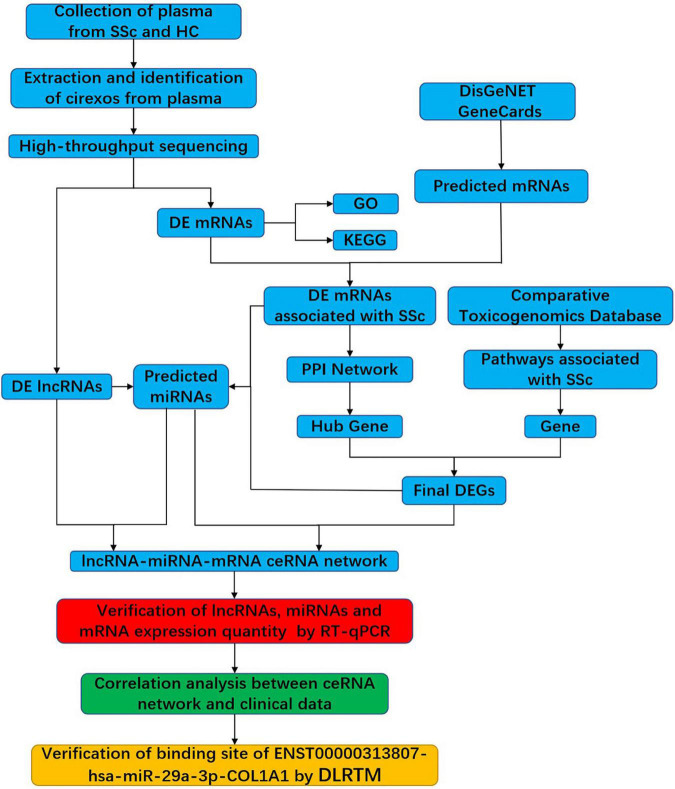
Flowchart of the experimental design. SSc, systemic sclerosis; HC, healthy control; cirexos, circulating exosomes; DEmRNAs, differentially expressed mRNAs; DElncRNAs, differentially expressed lncRNAs; DEGs, differentially expressed genes; GO, gene ontology; KEGG, Kyoto Encyclopedia of Genes and Genomes; PPI, protein–protein interaction; RT-qPCR, real-time quantitative PCR; DLRTM, dual-luciferase reporter.

In this study, cirexos were extracted and identified from the plasma of healthy controls (HCs) and patients with SSc. High-throughput sequencing and bioinformatics analysis were used to screen ceRNA networks in the SSc plasma cirexos. The ceRNA networks that were correlated with the clinical data of patients with SSc were verified with real-time quantitative PCR (RT-qPCR) and a double-luciferase reporter gene test (Figure 1). The results from this study provide potential biomarkers for the diagnosis and treatment of SSc.

## 2. Materials and methods

### 2.1. Patients and samples

Whole blood samples were collected from 20 patients with SSc and 20 age- and sex-matched HCs in the Department of Rheumatology and Physical Examination at the First Affiliated Hospital of Baotou Medical College, Inner Mongolia University of Science and Technology. The inclusion criteria of patients with SSc were in accordance with the 2013 American College of Rheumatology (ACR)/European League Against Rheumatism (EULAR) classification criteria. This study protocol was approved by the Ethics Committee of the First Affiliated Hospital of Baotou Medical College, Inner Mongolia University of Science and Technology [Approval No. 2018 (017)].

### 2.2. Extraction and identification of cirexos

Peripheral whole blood samples (8 mL) were collected with EDTA anticoagulant-coated tubes and then centrifuged at 3,000 rpm for 10 min at 25°C. The supernatant was divided into centrifuge tubes (Eppendorf, Hamburg, Germany) and stored at −80°C. The process was completed within 2 h. Cirexos were extracted with the miRNeasy serum/plasma kit (Qiagen, Hilden, Germany) according to the manufacturer’s instructions.

Precipitated cirexos were resuspended in radioimmunoprecipitation assay (RIPA) buffer (Solarbio, Beijing, China) to extract proteins. The protein concentration was determined using a BCA kit (Thermo Fisher Scientific, Waltham, MA, USA), and the cirexo protein markers were detected by western blot (WB). The protein was transferred to a polyvinylidene fluoride (PVDF) membrane following sodium dodecyl sulfate-polyacrylamide gel electrophoresis (SDS-PAGE). After blocking with 5% skimmed milk powder at 25°C for 2 h, specific primary antibodies against CD9 (1:5000), HRS (1:500) (Invitrogen, Camarillo, CA, USA), TSG101 (1:200), and calnexin (1:500) (Santa Cruz Biotechnology, San Diego, CA, USA) were incubated at 4°C for 8 h. The primary antibody was discarded the next day, and the secondary antibody (goat anti-rabbit HRP, 1:20,000) (Santa Cruz Biotechnology, CA, USA) was added and incubated at 37°C for 1 h. After incubating with a chemiluminescent substrate, the blot was photographed with an E-Gel imager (Thermo Fisher Scientific, Waltham, MA, USA). The particle size and distribution of cirexos were determined by a nanoparticle tracking analyzer (NTA) (ZetaView, Particle Matrix, Meersbusch, Germany). Analysis was performed using ZetaView 8.04.02 and Izon Control Suite 3.3.2 (IZON Science Ltd., Oxford, UK). In addition, transmission electron microscopy (TEM) (JEOL Ltd., Tokyo, Japan) was used to observe the isolated cirexos, and 10 mL of cirexos was used for electron microscopy detection and imaging at 100 kV.

### 2.3. RNA isolation and sequencing of cirexos

Prefiltered plasma was mixed with 2x binding buffer (XBP) at a ratio of 1:1 and then added to the exoEasy membrane affinity column. After centrifugation, the effluent was discarded, and washing buffer (XWP) was added to the column. After centrifugation, the flow through was discarded. QIAzol kit (QIAGEN, Hilden, Germany) was used to lyse the vesicles. Following centrifugation, the lysate was collected and chloroform was added and then thoroughly mixed. After centrifugation, ethanol was added to the aqueous phase. The sample–ethanol mixture was added onto RNeasy MinElute spin columns. After centrifugation, columns were washed once with buffer RWT, then twice with RPE buffer, before eluting RNA in water (15). The total RNA extracted from cirexos was subjected to high-throughput sequencing.

### 2.4. High-throughput screening of DEmRNAs and DElncRNAs

According to the *P*-value < 0.05 and | log2 (a Fold–change) | ≥ 1 standard, the differentially expressed mRNAs (DEmRNAs) and differentially expressed lncRNAs (DElncRNAs) in cirexos were obtained by high-throughput sequencing. The expression and clustering of DEmRNAs and DElncRNAs are presented by heatmaps and volcano maps. The mRNAs associated with SSc were predicted with the GeneCards database^1^ and DisGeNET database,^2^ and then the overlapping SSc-related genes and DEmRNAs were screened out and visualized by the online website Venn2.1.^3^

### 2.5. Analysis of gene ontology (GO) and Kyoto Encyclopedia of Genes and Genomes (KEGG) pathways

Gene ontology (GO) analysis of DEmRNAs was conducted by a Fisher’s exact test. The GO items with *P* < 0.05 were considered significantly enriched. Gene set enrichment analysis (GSEA, version 4.2.3)^4^ was used to perform GO analysis on all DEmRNAs obtained by sequencing. The random combination was set as 1,000 times. The gene matrix was set as c5.all.v7.5.1 symbols.gmt (GO), and GO items with a normalized enrichment score (NES) absolute value > 1 and *P* < 0.05 were screened. DEmRNAs were analyzed using the KEGG database with Fisher’s exact test. Pathways with a *P* < 0.05 were considered significantly enriched. The network of KEGG pathways was reconstructed by the ClueGO plug-in of Cytoscape3.9.1.^5^

### 2.6. Prediction of miRNAs targeted by DElncRNAs and DEmRNAs

lncRNASNP2^6^ was used to predict the miRNAs bound to DElncRNAs. Mature miRNA sequences from miRBase (21st edition) were collected in lncRNASNP2. To reduce false positives, the same miRNAs in MiRanda, TargetScan, and Pita were selected as the final target in lncRNASNP2 (16). miRNAs that interacted with SSc-associated DEmRNAs were predicted using Starbase v2.0.^7^ Data in lncRNASNP2 and Starbase v2.0 were supported by purple diplomatic immunoprecipitation and high-throughput sequencing experiments (17).

### 2.7. Prediction of lncRNA–miRNA–mRNA ceRNA networks

lncRNA–miRNA–mRNA networks were established according to the upregulated or downregulated mRNAs and lncRNAs in SSc, as well as the miRNAs that interacted with them. The STRING online tool^8^ was used to construct the protein–protein interaction (PPI) network of DEmRNAs. The minimum interaction score was set to be > 0.15. The data from the STING database were visualized and analyzed *via* Cytoscape 3.9.1 software. Hub genes in networks were identified with the cytoHubba plugin in Cytoscape 3.9.1 (18). Hub genes were calculated with three algorithms, including Maximal Clique Centrality (MCC), Degree, and Maximum Neighborhood Component (MNC), and then presented using the Venn diagram online tool (19, 20). The pathways related to SSc were predicted by the Comparative Toxicogenomics Database.^9^ The corresponding genes of the pathways were found, and the same DEmRNAs were used as the hub genes of the PPI networks. The lncRNA–miRNA–mRNA networks were drawn with Cytoscape3.9.1 software.

### 2.8. Prediction of mRNA and lncRNA localization

LncLocator^10^ is the prediction database of lncRNA subcellular localization, including localization of 15 cell lines (21, 22). The mRNALocator^11^ is a database for predicting mRNA subcellular localization (23). In this study, lncLocator and mRNALocator were used to predict the subcellular localization of lncRNA and mRNA, respectively.

### 2.9. Prediction of upstream transcription factors (TFs) and downstream binding proteins of lncRNAs

The binding sites of TFs for lncRNA were predicted by the ConSite database. The CatRAPID database^12^ was used to predict proteins that bind with lncRNA. PubMed^13^ was used to search for TFs and binding proteins related to SSc (24, 25).

### 2.10. ceRNA networks were verified by RT-qPCR

The relative expression levels of lncRNAs, miRNAs, and mRNAs involved in ceRNA networks from the plasma cirexo samples of 20 patients with SSc and 20 HCs were verified by RT-qPCR. The PCR cycling conditions were as follows: 95°C for 1 min; 40 cycles of 95°C for 15 s and 60°C for 30 s; and dissociation at 72 and 99°C. Data were analyzed by the 2-ΔΔCt method. Three technical replicates were used for each sample. The sequences of primers used in this study are listed in Table 1.

**TABLE 1:** Sequences of the primers used in this study.

Name	Sequence of primers (5′-3′)
ENST00000313807	Forward: GTGCTGGGTCGGGCTTCC
	Reverse: GTCGGGCGGCGGTCTTC
NONHSAT194388.1	Forward: TTAGCCAACATCACACTACTCCAAG
	Reverse: CCCACCTCAACCTCTCAAATAGC
COL1A1	Forward: AGGGCTGGGCGGGAGAG
	Reverse: ACACATCAAGACAAGAACGAGGTAG
hsa-miR-29a-3p	Stem-loop RT-primer: CTCAACTGGTGTCGTGGAGTCGGCAATTCAGTTGAGTAACCGAT
	Forward: ACACTCCAGCTGGGTAGCACCATCTGAAAT
hsa-miR-29b-3p	Stem-loop RT-primer: CTCAACTGGTGTCGTGGAGTCGGCAATTCAGTTGAGAACACTGA
	Forward: ACACTCCAGCTGGGTAGCACCATTTGAAATC
hsa-miR-29c-3p	Stem-loop RT-primer: CTCAACTGGTGTCGTGGAGTCGGCAATTCAGTTGAGTAACCGAT
	Forward: ACACTCCAGCTGGGTAGCACCATTTGAAAT

### 2.11. Correlation analysis between ceRNA networks and clinical data and receiver operating characteristic (ROC) curve drawing

The correlation between ceRNA networks was analyzed by IBM SPSS software Version 26.0 (SPSS Inc., Chicago, IL, USA) and GraphPad Prism 9.0.0 (GraphPad, La Jolla, CA, USA). Correlations between ceRNA networks and clinical data, such as high-resolution CT (HRCT), antinuclear antibody profile (Scl-70, CENP-B, Ro-52), C-reactive protein (CRP), IL-10, lymphocyte percentage, and neutrophil percentage, were analyzed, and then the receiver operating characteristic curve (ROC) for SSc diagnosis was drawn.

### 2.12. Verification of ceRNA interactions by a double-luciferase reporter gene assay

We synthesized the wild-type (WT) sequences of ENST00000313807 and *COL1A1* and cloned them into the pGL3 basic vector. According to the predicted interaction sites, the plasmid was used as a point-mutation template to construct a pGL3 basic vector. The pGL3 basic vector contains Firefly luciferase, and the pRL-TK plasmid contains Renilla luciferase, which was used as the control. The WT or mutation reporter plasmid was co-transfected with the hsa-miR-29a-3p mimic or negative control (NC) mimic into 293T cells. After 48 h, the luciferase activity of different groups was detected with the double-luciferase report gene detection kit (Beyotime, Shanghai, China). The relative light unit (RLU) values determined by firefly luciferase were divided by those determined by sea kidney luciferase. According to the ratio obtained, the activation degree of the target reporter gene was detected in different groups. The plasmids were divided into the following groups: NC mimics + ENST-WT; hsa-miR-29a-3p-mimics + ENST-WT; NC-mimics + ENST-mutant (MT); hsa-miR-29a-3p-mimics + ENST-MT; NC-mimics + *COL1A1*-WT; hsa-miR-29a-3p-mimics + *COL1A1*-WT; NC-mimics + *COL1A1*-MT; and hsa-miR-29a-3p-mimics + *COL1A1*-MT.

### 2.13. Statistical analysis

The data were analyzed by IBM SPSS software Version 26.0 (SPSS Inc., Chicago, IL, USA), GraphPad Prism 9.0.0 (GraphPad, La Jolla, CA, USA) and R Studio 4.1.0 (Boston, MA, USA). When the data of the correlation analysis were linear and conformed to normal distribution, Pearson correlation analysis was used for correlation analysis between ceRNA networks and clinical data; otherwise, Spearman’s correlation was used. When the data conformed to a normal distribution and the variance was homogeneous, an independe nt-sample *t* test was used; when the data conformed to a normal distribution and the variance was inhomogeneous, Welch’s *t* test was used; otherwise, a non-parametric test was used. Data are presented as the mean ± SEM, and tests were repeated three or five independent times. *P*-values < 0.05 were considered statistically significant. Binary logistic regression was also used for ROC curve analysis.

## 3. Results

### 3.1. Identification of plasma cirexos

**FIGURE 2:**
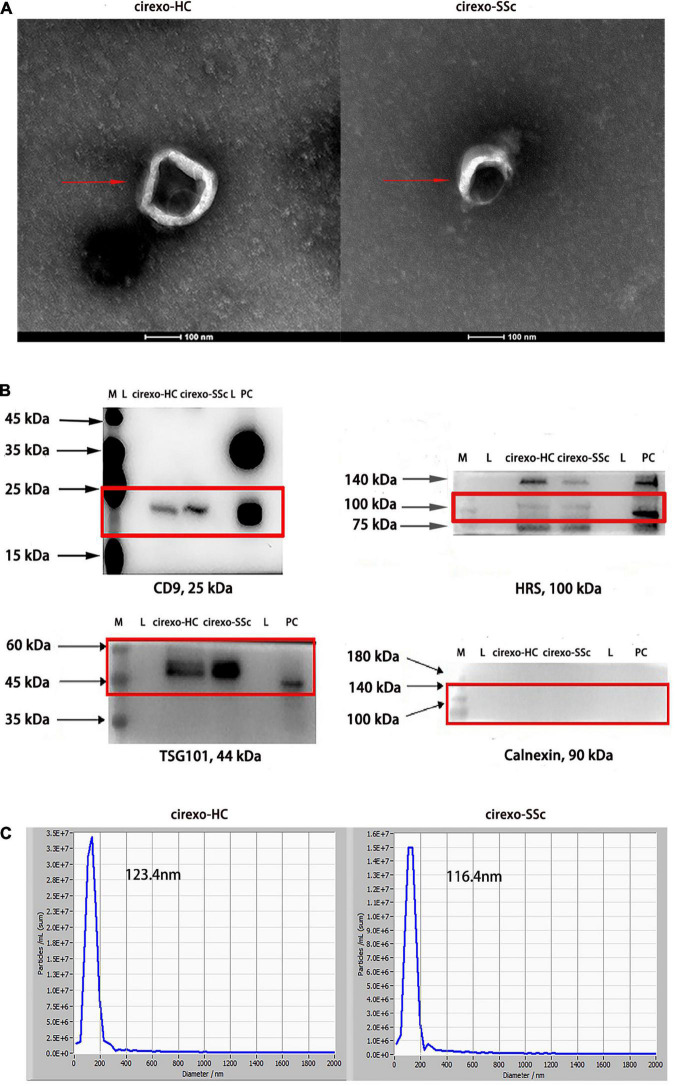
Identification of circulating exosomes (cirexos). **(A)** The morphology of cirexos was detected by transmission electron microscopy (TEM) (magnification: 300,000×). **(B)** Identification of cirexo surface markers by western blot (WB). M, marker; L, loading buffer; PC, exosomes (EXOs) derived from mouse macrophage supernatant, which was used as the positive control. **(C)** Determination of cirexo concentration and particle size by nanoparticle tracking analyzer (NTA). Cirexos, circulating exosomes; EXOs, exosomes.

In this study, TEM, WB, and NTA were used to identify the extracted cirexos. The results are shown in Figure 2. Clear vesicle structures were observed in plasma cirexos of SSc and HC by TEM, and the vesicle size was consistent with the detection standard of EXOs (Figure 2A). WB analysis showed the presence of the exosome markers hepatocyte growth factor-regulated tyrosine kinase substrate (HRS), CD9, and tumor susceptibility gene 101 (TSG101), but the absence of Calnexin (Figure 2B). The particle size of samples detected by NTA was consistent with the standard. The cirexo diameter of the HC sample was 123.4 nm, and the concentration was 1.2 × 10^11^ particles/mL. The cirexo diameter of the SSc sample was 116.4 nm, and the concentration of the sample was 9.5 × 10^10^ particles/mL (Figure 2C). The data indicated that cirexos were successfully extracted.

### 3.2. Analysis of DEmRNAs and DElncRNAs in plasma cirexos by high-throughput sequencing

**FIGURE 3:**
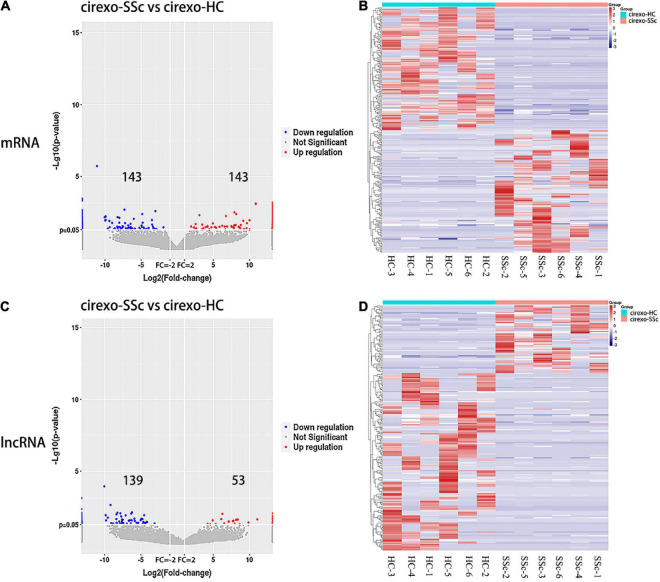
Expression profiles of mRNA and lncRNA in cirexos in the circulating exosomes–systemic sclerosis (cirexo-SSc) and circulating exosomes–healthy control (cirexo-HC) groups (*n* = 6). **(A,C)** Volcano plots show upregulated (red) and downregulated (blue) mRNA and lncRNA. The abscissa represents the fold change of gene expression in the different samples [log2 (fold-change)], and the ordinate represents the significance level of the differential gene expression [–log10 (*p*-value)]. **(B,D)** Heatmaps showing the hierarchical clustering analysis of differentially expressed mRNAs (DEmRNAs) and differentially expressed lncRNAs (DElncRNAs). The rows represent genes, and the columns represent the expression of the same sample. Red indicates high expression, and blue indicates low expression.

High-throughput sequencing was used to screen DEmRNAs and DElncRNAs in the plasma cirexos of SSc. The volcano map showed the overall distribution of differentially expressed genes (DEGs). The horizontal coordinate of the volcano map was the multiple of the difference, and a fold change greater than 2 was set to identify the differential genes. The ordinate was set as the minus base-10 logarithm of the *P*-value, which is -log 10 (*P*-value). The log10 (FPKM + 1) values were normalized and then clustered. The volcano map showed 286 DEmRNAs, including 143 upregulated genes and 143 downregulated genes (Figure 3A) (*P* < 0.05). The volcano map showed 192 DElncRNAs, including 53 upregulated lncRNAs and 139 downregulated lncRNAs (Figure 3C) (*P* < 0.05). The heatmap showed the expression levels of DEmRNAs (Figure 3B) and DElncRNAs (Figure 3D) in cirexos from the SSc and HC groups. The selected 286 DEmRNAs were used for further GO and KEGG enrichment analysis to explore the potential biological functions of DEmRNAs and the corresponding enrichment pathways.

### 3.3. GO enrichment analysis of DEmRNAs in cirexo

**FIGURE 4:**
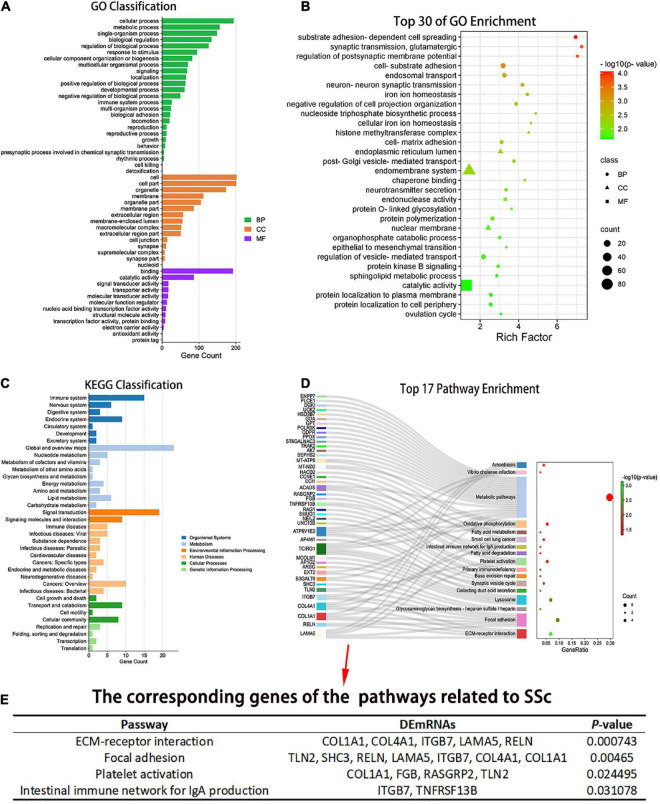
Gene ontology (GO) and Kyoto Encyclopedia of Genes and Genomes (KEGG) enrichment analysis of the DEmRNAs. **(A)** The number of differentially expressed genes (DEGs) corresponding to the three GO levels, including biological process (BP), cellular component (CC), and molecular function (MF), was counted. **(B)** Top 30 GO entries with enrichment degree. The ordinate is the specific GO entry name. **(C)** KEGG classification. The abscissa is the number of genes mapped to a pathway class by DEGs, and the ordinate is the KEGG entries enriched by DEGs. **(D)** Pathway enrichment of the differential mRNAs. The abscissa is the proportion of genes corresponding to a certain pathway from the corresponding genes of all pathways. The ordinate is the KEGG entry name with *P* < 0.05 and the DEGs corresponding to the enrichment pathway. **(E)** Pathways related to SSc and corresponding genes with *P* < 0.05. GO, gene ontology; KEGG, Kyoto Encyclopedia of Genes and Genomes; BP, biological process; CC, cellular component; MF, molecular function.

The number of DEmRNAs corresponding to the cell component (CC), molecular function (MF), and biological process (BP) of GO was counted and graphically displayed. In biological processes, DEmRNA mainly focuses on cellular process, metabolic process, single biological process, biological regulation, regulation of biological process, response to stimulus, cellular component organization and biogenesis, multicellular biological process, signal, and localization. In cellular components, DEmRNAs were mainly concentrated in the cell, cell part, organelle, membrane, organelle part, membrane part, extracellular region, membrane closed cavity, polymer complex, and extracellular region part. Among the molecular functions, binding and catalytic activities were mainly enriched (Figure 4A). There were 67 GO biological pathways with a *P*-value < 0.05, including 47 biological process pathways, 11 cellular-component pathways, and 9 molecular-function pathways. Pathways related to SSc were screened through the Comparative Toxicogenomics Database. Among the top 30 enriched pathways, the pathways related to SSc mainly included matrix adhesion-dependent cell spreading, post-Golgi vesicle-mediated transport, epithelial–mesenchymal transition (EMT), endonuclease activity, intracellular transport, cell-matrix adhesion, and other processes (Figure 4B).

### 3.4. Analysis of pathway enrichment of DEmRNAs in cirexos

Pathway analysis of DEmRNAs was performed on the KEGG database using Fisher’s exact test. DEmRNAs in cirexos of patients with SSc were mainly enriched in immune system, immune diseases, signal transduction, lipid metabolism, cell growth and apoptosis, and cell movement (Figure 4C). Seventeen pathways had a *P*-value < 0.05 (Figure 4D). The pathways related to SSc were screened through the Comparative Toxicogenomics Database. The pathways related to SSc and corresponding DEmRNAs are shown in Figure 4E, including extracellular matrix (ECM) receptor interaction, focal adhesion, platelet activation, and intestinal immune network for IgA production (Figure 4E).

**FIGURE 5:**
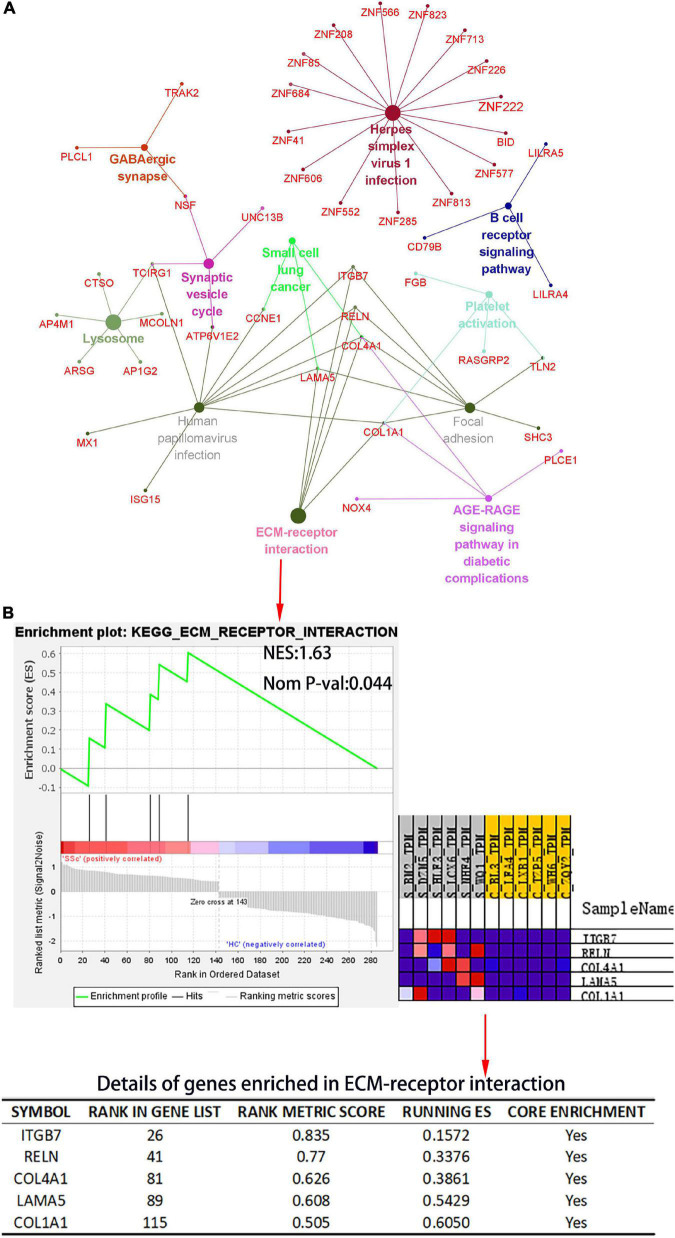
Kyoto Encyclopedia of Genes and Genomes (KEGG) pathway analysis of the DEmRNA and gene set enrichment analysis (GSEA) of differential gene expression profiles by c2, curated gene sets. **(A)** KEGG pathway analysis of DEmRNAs. The plug-in ClueGO of Cytoscape was used to map KEGG pathway interactions. Different colors represent different pathways. **(B)** The c2: KEGG gene set was used to conduct pathway enrichment analysis of DEmRNA profiles. In the table, “RANK IN GENE LIST” represents the position of a gene in the sorted gene set, “RANK METRIC SCORE” is the ranking score of genes, “RUNNING ES” is the dynamic enrichment score (ES) value in the analysis process, and “CORE ENRICHMENT” is the gene that mainly contributes to the ES value. NES, normalized enrichment score; Nom *P*-val, nominal *P*-value.

The Cytoscape plug-in ClueGO was used to analyze pathway enrichment of the DEmRNAs, and a total of 11 significantly enriched pathways were obtained. Pathways related to SSc were screened using the Comparative Toxicogenomics Database. ECM receptor interaction, focal adhesion, and platelet activation were associated with SSc. *TCIRG1, ITGB7, RELN, COL4A1, LAMA5*, and *COL1A1* were enriched in more than three pathway terms (Figure 5A). GSEA was used to analyze the pathway enrichment of DEmRNAs. The ECM-receptor interaction gene set showed the leading subset in the enrichment score (ES) diagram, with the absolute value of NES > 1, *P* < 0.05, indicating that this functional gene set had significant biological significance. *ITGB7, RELN, COL4A1, LAMA*, and *COL1A1* played core roles in the curated gene sets. The heatmap shows that the expression of these genes was upregulated in SSc (*P* < 0.05) (Figure 5B).

### 3.5. Prediction of miRNAs targeted by DElncRNAs and DEmRNAs

**FIGURE 6:**
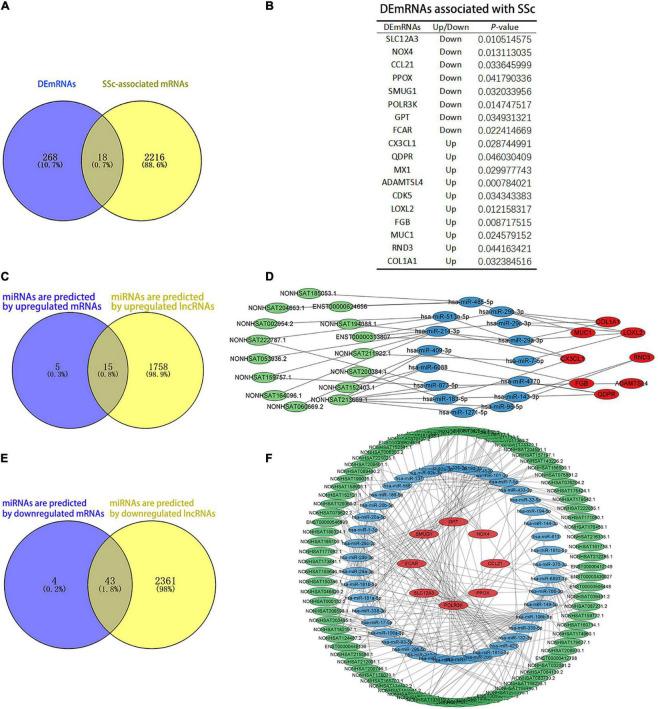
Analysis of competing endogenous RNAs (ceRNAs) networks. **(A)** The blue and yellow parts are Venn diagrams. The blue part shows the DEmRNAs obtained by sequencing. The yellow part is the mRNAs predicted by the DisGeNET and GeneCards databases, and the middle part represents the overlapping DEmRNAs of the two groups. **(B)** The table of DEmRNAs related to SSc. **(C,D)** Upregulated ceRNA network. Panel **(C)** the blue and yellow parts are Venn diagrams. The blue part shows the miRNAs that are predicted by upregulated mRNAs. The yellow part shows the miRNAs that are predicted by upregulated lncRNAs. Panel **(D)** green parts are upregulated lncRNA, blue parts are miRNAs that interact with lncRNA and mRNA, and red parts are upregulated mRNA. **(E,F)** Downregulated ceRNA network. Panel **(E)** the blue and yellow parts are Venn diagrams. The blue part shows the miRNAs predicted by downregulated mRNAs. The yellow part shows the miRNAs predicted by downregulated lncRNAs. Panel **(F)** the green parts are downregulated lncRNA, blue parts are miRNAs that interact with lncRNA and mRNA, and red parts are downregulated mRNA.

The results showed that 2,234 mRNAs were associated with SSc, as predicted by the Genecards and DisGeNET databases. We found 18 DEmRNAs that overlapped with mRNAs associated with SSc as predicted by the databases (Figure 6A). Among them, 10 were upregulated and 8 were downregulated (Figure 6B). ENCORI was used to predict the miRNAs bound to upregulated and downregulated DEmRNAs. LncRNASNP2 was used to predict the miRNAs bound to upregulated and downregulated DElncRNAs. The results showed that there were 20 miRNAs that interacted with the upregulated DEmRNAs, and 1,773 miRNAs that interacted with upregulated DElncRNAs, 15 of which were the same miRNAs (Figure 6C). Moreover, there were 47 miRNAs that interacted with downregulated DEmRNAs, and 2,404 miRNAs that interacted with downregulated DElncRNAs, 43 of which were the same miRNAs (Figure 6E). In addition, 15 upregulated lncRNAs, 15 miRNAs, and 8 upregulated mRNAs were obtained to construct ceRNA networks (Figure 6D), and 85 downregulated lncRNAs, 43 miRNAs, and 8 downregulated mRNAs were obtained to construct the ceRNA networks (Figure 6F).

### 3.6. Hub genes were screened by PPI networks

**FIGURE 7:**
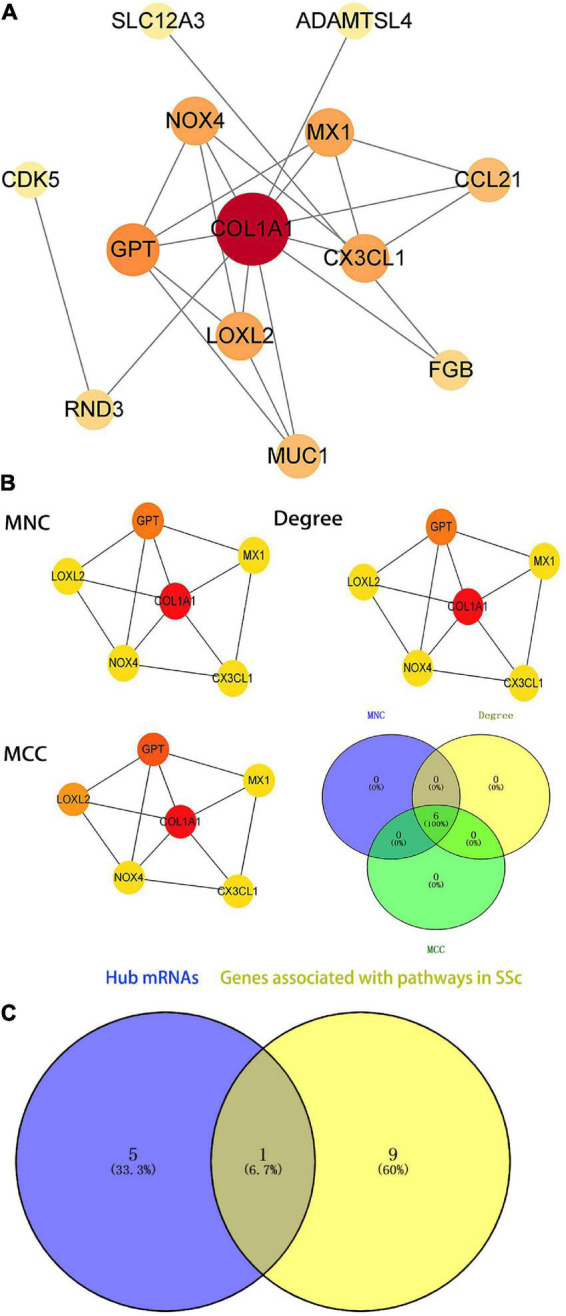
Identification of the hub genes in the protein–protein interaction (PPI) network. **(A)** PPI network of differentially expressed genes (DEGs) related to systemic sclerosis (SSc). The ceRNA networks of DEG encoded proteins are composed of 18 nodes and 19 edges. Each node represents a protein, and each edge represents a protein-protein association. **(B)** Identification of the hub genes of DEGs associated with SSc in the PPI network. The hub genes are identified by three algorithms: Maximal Clique Centrality (MCC), Degree, and Maximum Neighborhood Component (MNC). The color density represents the score of the algorithm. **(C)** The blue part presents the hub genes of the PPI network. The yellow part shows the genes associated with SSc-related pathways (*P* < 0.05 in KEGG). The middle part represents the overlapping hub genes of the two groups. MCC, maximal clique centrality; MNC, maximum neighborhood component.

To further illustrate the interactions between selected DEGs, the STRING online tool was used to form a PPI network. Protein interaction analysis of DEmRNAs was performed with the STRING online tool. PPI networks were constructed with the lowest interaction score > 0.15 and visualized in Cytoscape (Figure 7A). The top six hub genes were calculated according to MCC, Degree, and MNC algorithms (Figure 7B), and included *COL1A1, CX3CL1, LOXL2, GPT, NOX4*, and *MX1*. These genes were the most important genes in the PPI network and may contribute to the pathogenesis of SSc. *COL1A1* was a hub gene in the PPI network and also in the SSc-related pathway *via* KEGG analysis, with a *P*-value < 0.05 (Figure 7C). Therefore, in the follow-up study, we will focus on the screened hub gene, namely *COL1A1*.

### 3.7. GO enrichment analysis of DEG expression profiles by the GSEA c5 gene set

**FIGURE 8:**
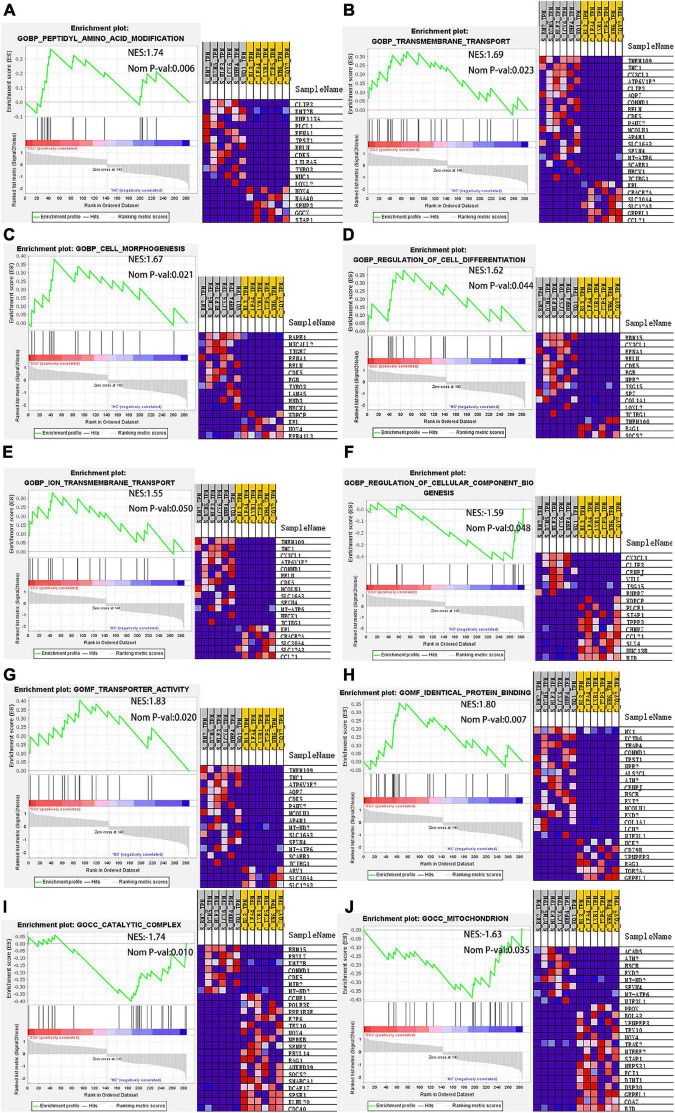
GSEA. In BP, the gene sets with significantly enriched upregulated genes include **(A)** peptidyl amino acid modification, **(B)** transmembrane transport, **(C)** cell morphogenesis, **(D)** regulation of cell differentiation, and **(E)** ion transmembrane transport. The gene set with significantly enriched downregulated genes includes **(F)** regulation of cellular component biogenesis. In MF, the gene sets with significantly enriched upregulated genes include **(G)** transporter activity and **(H)** identical protein binding. In CC, the gene sets with significant enrichment of downregulated genes include **(I)** catalytic complex mitochondrion and **(J)** mitochondrion. GSEA, gene set enrichment analysis; BP, biological process; MF, molecular function; CC, cell component.

Next, GO enrichment analysis of the DEG expression profile was performed by GSEA to further explore the biological function of DEGs. The DEmRNAs obtained by high-throughput sequencing were uploaded to GSEA, and the expression profiles were analyzed with c5 (GO gene sets). GO items with a NES absolute value > 1 and *P* < 0.05 were screened. The significantly upregulated gene sets in the BP include peptidyl amino acid modification, transmembrane transport, cell morphogenesis, regulation of cell differentiation, and ion transmembrane transport. The significantly downregulated gene sets include regulation of cellular component biogenesis. In MF, the gene sets with significantly enriched upregulated genes were transporter activity and identical protein binding. In CC, the gene sets of significantly enriched downregulated genes were catalytic complex mitochondrion and mitochondrion. Genes that played a central role in the above sets, including *COL1A1, NOX4*, and *CX3CL1*, were the same as the hub genes in the PPI network. According to our results, *NOX4*, which interacts with *COL1A1*, plays a central role in the cellular complex mitochondrion. *CX3CL1*, which interacts with *COL1A1*, plays a central role in transport, regulation of cell differentiation, and ion transport. *COL1A1* is involved in the regulation of cell differentiation in BP and is associated with conspecific protein binding in MF. *NOX4* is involved in protein modification and cell morphology in BP and is related to catalysis and mitochondria in CC. *CX3CL1* is involved in the regulation of cell differentiation, ion transmembrane transport, and biogenesis of cellular components in BP (Figure 8). The results showed that *COL1A1* plays a key role in GO enrichment. Therefore, in this study, ceRNA networks were established for further analysis of *COL1A1*.

### 3.8. Prediction of CeRNA networks and the lncRNA–miRNA–mRNA interaction sites

**FIGURE 9:**
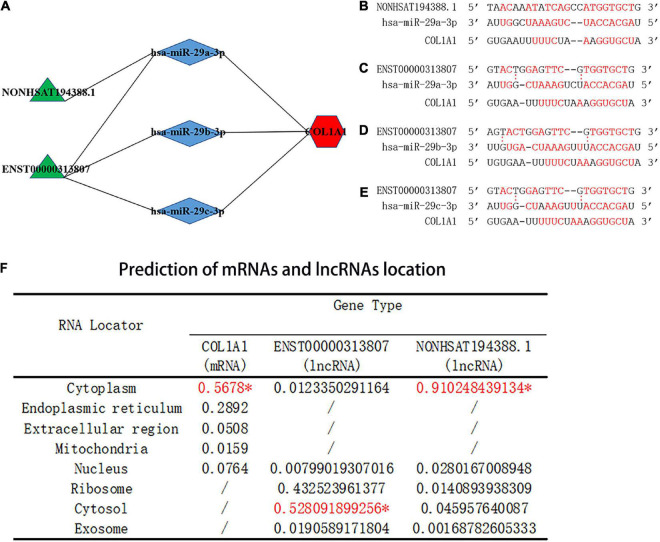
Prediction of the binding sites and location of the ceRNA networks. **(A)** Prediction of ceRNA networks. Green parts are lncRNA, blue parts are miRNAs that interact with lncRNA and mRNA, and red parts are mRNA. **(B)** The red text represents binding sites of the NON-HSAT194388.1-hsa-miR-29a-3p-*COL1A1* interaction network. **(C)** The red text represents binding sites of the ENST00000313807-hsa-miR-29a-3p-*COL1A1* interaction network. **(D)** The red text represents binding sites of the ENST00000313807-hsa-miR-29b-3p-*COL1A1* interaction network. **(E)** The red text represents binding sites of the ENST00000313807-hsa-miR-29c-3p-*COL1A1* interaction network. “:” Means that the bond is not firm, and the red font means that the bond is stable. **(F)** Prediction of miRNA and lncRNA localization. The “*” indicates the final predicted position on the website.

Salmena et al. (26) proposed a ceRNA hypothesis suggesting that, due to the existence of miRNA response elements (MREs) on mRNA and lncRNA, miRNAs can bind to target mRNA and lncRNA to post-transcriptionally regulate gene expression. As complementary sequences of miRNAs, mRNA and lncRNA form a large-scale regulatory network in various parts of the transcriptome (26). Based on this hypothesis, mRNA or lncRNA binds to miRNA, forming a competitive relationship. In this study, the hub gene *COL1A1* was upregulated in SSc (*P* < 0.05). According to the ENCORI database, the predicted miRNAs that interacted with *COL1A1* included hsa-miR-29a-3p, hsa-miR-29b-3p, and hsa-miR-29c-3p. The predicted lncRNAs that interacted with hsa-miR-29a-3p, hsa-miR-29b-3p, and hsa-miR-29c-3p included ENST00000313807 and NON-HSAT194388.1. Therefore, four ceRNA networks were constructed (Figure 9A), and the lncRNA–miRNA–mRNA interaction sites were predicted (Figures 9B–E).

### 3.9. Prediction of mRNA and lncRNA localization

The subcellular localization of lncRNAs involved in ceRNA is crucial for the study of ceRNAs. As the main location of ceRNAs is the cytoplasm, lncRNAs involved in ceRNAs need to be expressed in the cytoplasm to regulate the expression of targeted mRNAs (27, 28). Cytoplasmic lncRNAs play key roles in the cell through various molecular mechanisms, including regulating the transport of cytoplasmic proteins from the cytosol to nucleus to regulate transcription (28–30). Based on the prediction score in the mRNALocater online database, *COL1A1* was predicted to be located in the cytoplasm. Based on the prediction score, ENST00000313807 and NON-HSAT194388.1 were predicted to be located in the cytoplasm by the lncLocater online database (Figure 9F). The results suggested that *COL1A1*, ENST00000313807, and NON-HSAT194388.1 were consistent with the mechanism of ceRNA in the cytoplasm.

### 3.10. Prediction of upstream TFs and downstream binding proteins of lncRNAs

**FIGURE 10:**
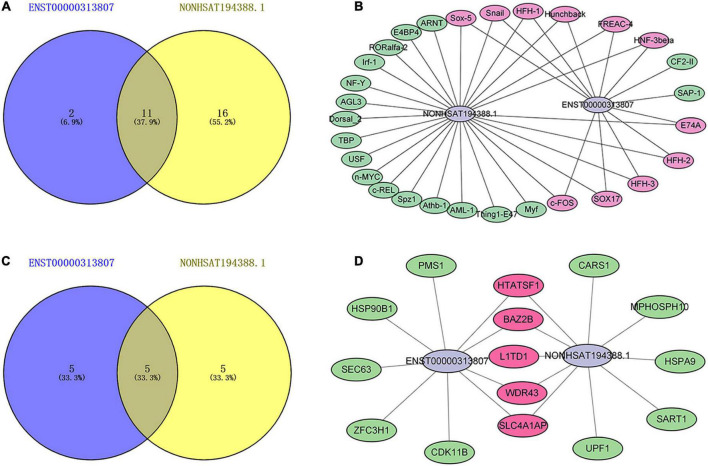
Maps of lncRNAs binding to upstream transcription factors (TFs) and downstream binding proteins. **(A)** A Venn diagram of TFs binding to ENST00000313807 and NON-HSAT194388.1. The blue part shows the TFs binding to ENST00000313807. The yellow part shows the TFs binding to NON-HSAT194388.1, and the middle part represents the overlapping TFs of the two groups. **(B)** Binding map of lncRNAs and upstream TFs. The green part shows the TFs binding to ENST00000313807 or NON-HSAT194388.1. The purple part represents the overlapping TFs of the two groups. **(C)** A Venn diagram of proteins binding to ENST00000313807 and NON-HSAT194388.1. The blue part shows the proteins binding to ENST00000313807. The yellow part shows the proteins binding to NON-HSAT194388.1, and the middle part represents the overlapping proteins of the two groups. **(D)** Binding map of lncRNAs and downstream proteins. The green part shows the proteins binding to ENST00000313807 or NON-HSAT194388.1. The purple part represents the overlapping proteins of the two groups.

Transcription factors (TFs) can regulate the expression of lncRNA by binding to its promoter region (31). The upstream TFs of ENST00000313807 and NON-HSAT194388.1 were predicted by the database ConSite. The TFs that overlapped between both groups included E74A, c-FOS, Hunchback, Sox-5, FREAC-4, Snail, HFH-2, HNF-3beta, HFH-1, SOX17, and HFH-3. Among them, the TFs related to SSc were c-FOS, Snail, and SOX17, as identified from the PubMed database (32–35; Figures 10A, B). Interaction with binding proteins is an important function of lncRNAs. We predicted the downstream binding proteins of ENST00000313807 and NON-HSAT194388.1 through the online website catrapid, and selected the top 10 scores of predicted binding ability. The following five binding proteins were obtained: SLC4A1AP, L1TD1, WDR43, HTATSF1, and BAZ2B (Figures 10C, D). However, an extensive literature search involving these proteins in the PubMed database did not reveal any studies related to SSc.

### 3.11. Validation of RNA-Seq data by RT-qPCR and ROC curve

**FIGURE 11:**
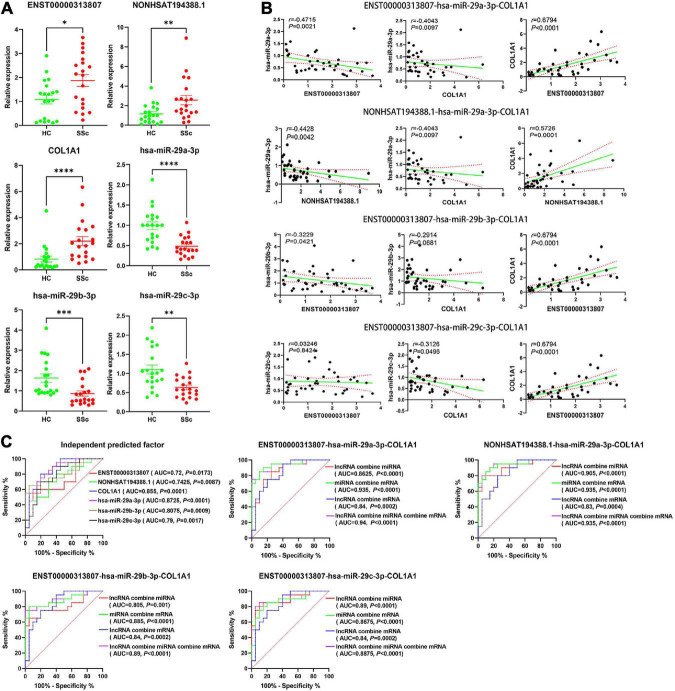
**(A)** The relative expression of ENST00000313807, NON-HSAT194388.1, *COL1A1*, hsa-miR-29a-3p, hsa-miR-29b-3p, and hsa-miR-29c-3p in plasma cirexos was verified by Real-time Quantitative PCR (RT-qPCR) (*n* = 20). The green part shows the relative expression of RNA in healthy control; (HCs). The red part shows the relative expression of RNA in patients with SSc. *P < 0.05, ^**^P < 0.01, ^***^P < 0.001, and ^****^P < 0.0001. **(B)** Correlation analysis of the ENST00000313807-hsa-miR-29a-3p-*COL1A1* networks, NON-HSAT194388.1-hsa-miR-29a-3p-*COL1A1* networks, ENST00000313807-hsa-miR- 29b-3p-*COL1A1* networks, and ENST00000313807-hsa-miR-29c-3p-*COL1A1* networks found in plasma cirexos (*n* = 20). The solid green line is a regression straight line, which is used to describe the relationship curve between the dependent variable y and the independent variable x with a linear relationship. The area formed by the red dotted line represents the 95% confidence interval. **(C)** Receiver operating characteristic (ROC) curve analysis of the independent diagnosis of ceRNA networks, ENST00000313807-hsa-miR-29a-3p-*COL1A1* combined diagnosis, NON-HSAT194388.1-hsa-miR-29a- 3p-*COL1A1* combined diagnosis, ENST00000313807-hsa-miR-29b-3p-*COL1A1* combined diagnosis, and ENST00000313807-hsa-miR-29c-3p-*COL1A1* combined diagnosis in plasma cirexos (*n* = 20). AUC, area under curve.

To detect the expression of RNA in the ceRNA networks predicted above, plasma cirexos in 20 SSc and 20 HC cases were detected by RT-qPCR. Compared to the cirexo-HC group, the expression levels of ENST00000313807 (*P* < 0.05), NON-HSAT194388.1 (*P* < 0.05), and *COL1A1* (*P* < 0.01) in the SSc-cirexo group were significantly increased, while the expression levels of hsa-miR-29a-3p (*P* < 0.0001), hsa-miR-29b-3p (*P* < 0.01), and hsa-miR-29c-3p (*P* < 0.01) were significantly decreased (Figure 11A). ENST00000313807, NON-HSAT194388.1, and *COL1A1* were upregulated in cirexos isolated from the plasma of patients with SSc. These results were consistent with the high-throughput sequencing data.

The correlation analysis of the lncRNA–miRNA–mRNA networks was analyzed. The results showed that ENST00000313807 and NON-HSAT194388.1 were negatively correlated with hsa-miR-29a-3p (*P* < 0.01). ENST00000313807 (*P* < 0.0001), and NON-HSAT194388.1 (*P* = 0.0001) were positively correlated with *COL1A1*, and hsa-miR-29a-3p was negatively correlated with *COL1A1* (*P* < 0.01). lncRNAs involved in the ceRNA mechanism are positively correlated with mRNAs, while lncRNAs and mRNAs are negatively correlated with miRNAs (10, 36). The relationships of ENST00000313807-hsa-miR-29a-3p-*COL1A1* and NON-HSAT194388.1-hsa-miR-29a-3p-*COL1A1* conformed to the ceRNA mechanism. ENST00000313807 was negatively correlated with hsa-miR-29b-3p (*P* < 0.05), while *COL1A1* was not significantly correlated with hsa-miR-29b-3p (*P* > 0.05). There was no significant correlation between ENST00000313807 and hsa-miR-29c-3p (*P* > 0.05), while *COL1A1* was negatively correlated with hsa-miR-29c-3p (*P* < 0.05) (Figure 11B). Therefore, the relationships of ENST00000313807-hsa-miR-29b-3p-*COL1A1* and ENST00000313807-hsa-miR-29c-3p-*COL1A1* did not conform to the ceRNA mechanism.

The ROC curve was used for independent or combined diagnosis of SSC. The results showed that the independent diagnostic value of hsa-miR-29a-3p was higher than that of the others [area under curve (AUC): 0.8725, cutoff: 0.9115]. The top combined diagnosis ceRNA networks were as follows: ENST00000313807-hsa-miR-29a-3p-*COL1A1* (AUC: 0.94, *P* < 0.0001), NON-HSAT194388.1-hsa-miR-29a- 3p-*COL1A1* (AUC: 0.935, *P* < 0.0001), hsa-miR-29a-3p-*COL1A1* (AUC: 0.935, *P* < 0.0001), ENST00000313807-hsa-miR-29b-3p-*COL1A1* (AUC: 0.89, *P* < 0.0001), and ENST00000313807-hsa-miR-29a-3p (AUC: 0.89, *P* < 0.0001) (Figure 11C). Therefore, ENST00000313807, NON-HSAT194388.1, *COL1A1*, hsa-miR-29a-3p, hsa-miR-29b-3p, and hsa-miR-29c-3p may be used as biomarkers for the diagnosis of SSc. The combined diagnosis is more valuable than independent diagnosis.

### 3.12. Correlation analysis between ceRNA networks and clinical data

**FIGURE 12:**
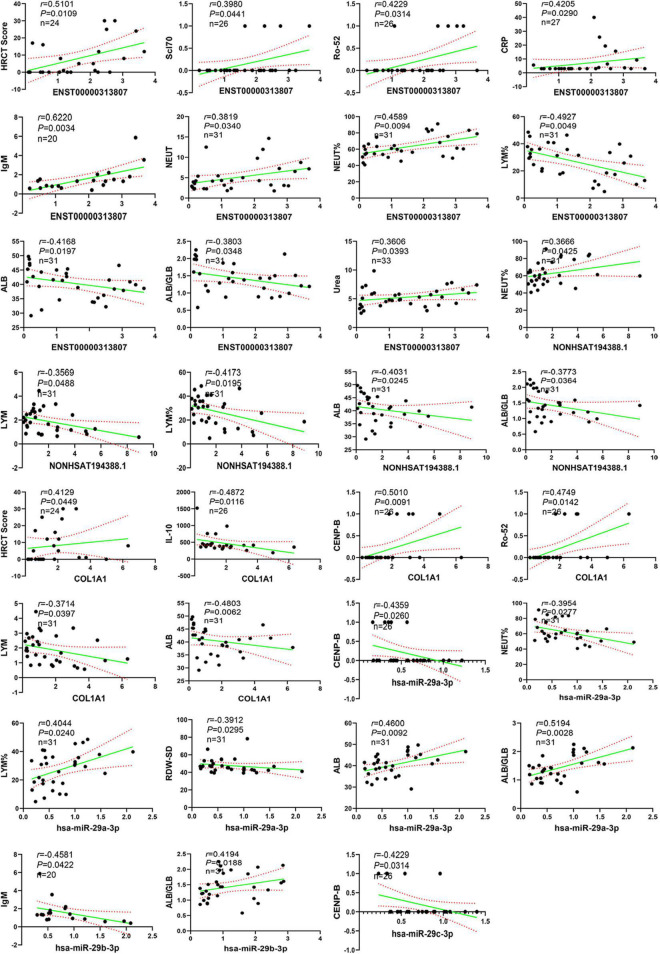
Correlation analysis between RNA expression and clinical data. The solid green line is a regression straight line, which is used to describe the relationship curve between the dependent variable y and the independent variable x with a linear relationship. The area formed by the red dotted line represents the 95% confidence interval. NEUT, neutrophil; NEUT%, neutrophil percentage; LYM, lymphocytes; LYM%, lymphocytes percentage; ALB, albumin; ALB/GLB, albumin divided by globulin; RDW-SD, red cell distribution width-standard deviation; HRCT, high-resolution CT; CRP, C-reactive protein.

**FIGURE 13:**
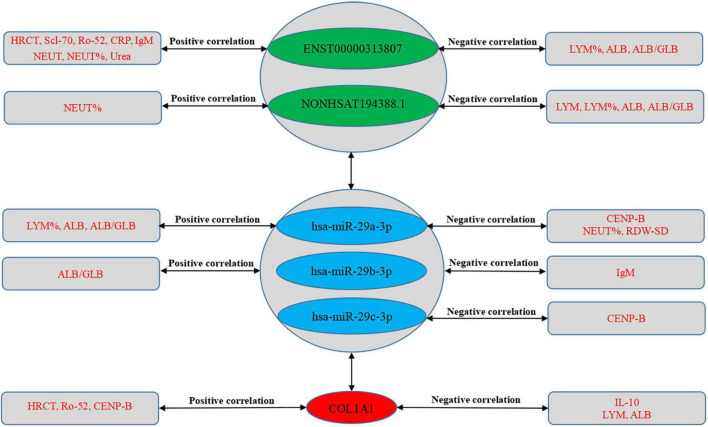
Correlation between RNA expression and clinical data. The green part shows lncRNAs, the blue part shows miRNAs, and the red part shows mRNAs in the middle of the figure. The text in the rectangles on both sides of the figure is clinical data.

We further evaluated the relationship between ceRNA networks in the plasma cirexos and the clinical features of 20 patients with SSc. The results showed that ENST00000313807 was positively correlated with HRCT score (*r* = 0.5101, *P* = 0.0109), Scl-70 (*r* = 0.398, *P* = 0.0441), Ro-52 (*r* = 0.4229, *P* = 0.0314), CRP (*r* = 0.4205, *P* = 0.4205), IgM (*r* = 0.622, *P* = 0.0034), neutrophil count (NEUT) (*r* = 0.3819, *P* = 0.034), neutrophil percentage (NEUT%) (*r* = 0.4589, *P* = 0.0094), and urea (*r* = 0.3606, *P* = 0.0393). ENST00000313807 was negatively correlated with lymphocyte percentage (LYM%) (*r* = −0.4927, *P* = 0.0049), albumin (ALB) (*r* = −0.4168, *P* = 0.0197), and white sphere ratio (ALB/GLB) (*r* = −0.3803, *P* = 0.0348). The results showed that NON-HSAT194388.1 was positively correlated with NEUT% (*r* = 0.3666, *P* = 0.0425), and NON-HSAT194388.1 was negatively correlated with LYM (*r* = −0.3569, *P* = 0.0488), LYM% (*r* = −0.4173, *P* = 0.0195), ALB (*r* = −0.4031, *P* = 0.0245), and ALB/GLB (*r* = −0.3773, *P* = 0.0364). Furthermore, *COL1A1* was positively correlated with HRCT score (*r* = 0.4129, *P* = 0.0449), Ro-52 (*r* = 0.4749, *P* = 0.0142), and CENP-B (*r* = 0.501, *P* = 0.0091), and *COL1A1* was negatively correlated with IL-10 (*r* = −0.4872, *P* = 0.0116), ALB (*r* = −0.4803, *P* = 0.0062), and LYM (*r* = −0.3714, *P* = 0.0397). The results showed that hsa-miR-29a-3p was positively correlated with LYM% (*r* = 0.4044, *P* = 0.024), ALB (*r* = 0.46, *P* = 0.0092), and ALB/GLB (*r* = 0.5194, *P* = 0.0028), and hsa-miR-29a-3p was negatively correlated with CENP-B (*r* = −0.4359, *P* = 0.026), NEUT% (*r* = −0.3954, *P* = 0.0277), and the standard deviation of red blood cell distribution width (RDW-SD) (*r* = −0.3912, *P* = 0.0295). Moreover, hsa-miR-29b-3p was positively correlated with ALB/GLB (*r* = 0.4194, *P* = 0.0188), and hsa-miR-29b-3p was negatively correlated with IgM (*r* = −0.4581, *P* = 0.0422). The results showed that hsa-miR-29c-3p was negatively correlated with CENP-B (*r* = 0.4229, *P* = 0.0314) (Figures 12, 13). Therefore, ENST00000313807, NON-HSAT194388.1, *COL1A1*, hsa-miR-29a-3p, hsa-miR-29b-3p, and hsa-miR-29c-3p in ceRNA networks may be used as a diagnostic biomarker or therapeutic target for SSc in the future. It is worth noting that the greatest correlation was observed between the clinical data and the ENST00000313807-hsa-miR-29a-3p-*COL1A1* network. Thus, the ENST00000313807-hsa-miR-29a-3p-*COL1A1* network could have the most potential as a combined biomarker to diagnose SSc.

### 3.13. Verification of the ENST00000313807-hsa-miR-29a-3p-*COL1A1* interaction by a double-luciferase reporter gene assay

**FIGURE 14:**
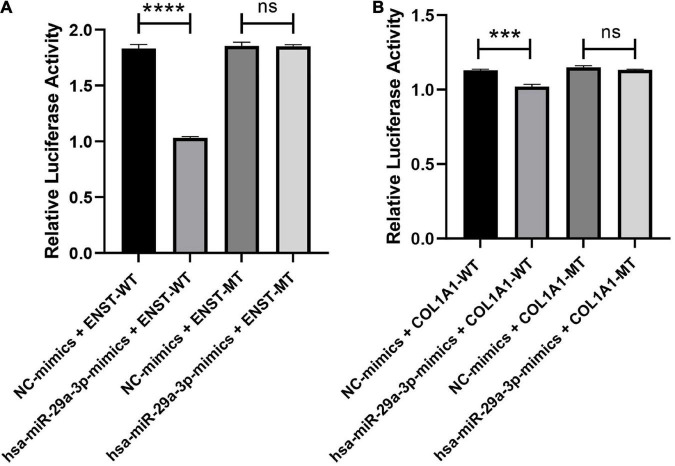
Verification of the ENST00000313807-hsa-miR-29a-3p-*COL1A1* interaction by a double-luciferase reporter gene assay. **(A)** The WT or mutation reporter plasmid of ENST00000313807 was co-transfected with the hsa-miR-29a-3p mimic or negative control (NC) mimic into 293T cells. **(B)** The WT or mutation reporter plasmid of *COL1A1* was co-transfected with the hsa-miR-29a-3p mimic or negative control (NC) mimic into 293T cells. ****P < 0.0001, ***P < 0.001. NC, negative control; WT, wild type; MT, mutant; ns, no significance.

The interaction of ENST00000313807-miR-29a-3p-*COL1A1* in plasma cirexos was detected by a double-luciferase reporter gene test. The results showed that the fluorescence value of the hsa-miR-29a-3p-mimics + ENST-WT group was significantly lower than that of the NC mimics + ENST-WT group (*P* < 0.0001). There was no significant difference in the fluorescence value between the hsa-miR-29a-3p-mimics + ENST-MT group and the NC mimics + ENST-MT group (*P* > 0.05). Compared to the NC mimics + *COL1A1*-WT group, the fluorescence value of the hsa-miR-29a-3p-mimics + *COL1A1*-WT group decreased significantly (*P* < 0.0001). There was no significant difference in the fluorescence value between the hsa-miR-29a-3p-mimics + *COL1A1*-MT group and the NC mimics + *COL1A1*-MT group (*P* > 0.05) (Figure 14). The results showed an interaction between ENST00000313807 and hsa-miR-29a-3p, and *COL1A1* and hsa-miR-29a-3p in plasma cirexos. Therefore, the ENST00000313807-hsa-miR-29a-3p-*COL1A1* network can not only be used as a combined biomarker to diagnose SSc, but ENST00000313807 also interacts with hsa-miR-29a-3p, which interacts with *COL1A1*. However, whether the ENST00000313807-hsa-miR-29a-3p-*COL1A1* network participates in the biological process of SSc pathogenesis through interaction, as well as the underlying mechanism, requires further study.

## 4. Discussion

At present, there are few specific markers for SSc, and their sensitivity is low. Most patients with SSc can be diagnosed only after they have had organ damage in the middle and late stages of disease. Biomarkers with high specificity and sensitivity are needed for the diagnosis of SSc in the early stage of disease development (37). Wu et al. (38) analyzed the differentially expressed miRNAs (DEmiRNAs) and DEmRNAs in the lung tissues of patients with SSc-ILD and HCs. The core mRNAs in the ceRNA networks included *COL1A1*, endothelin (*EDN1*) and fos proto-oncogene (*FOS*). The expression of *COL1A1* had a negative relationship with central granulocyte. The expression of *FOS* was associated with increased mast cells. The expression of *EDN1* had a positive relationship with the number of mast cells and natural killer cells. Yan et al. (39) analyzed the DEmRNAs and DEmiRNAs between lung tissues in SSc-ILD and HCs, and predicted lncRNAs and mRNAs binding to miRNAs using online databases. They found that ceRNA networks, such as LINC01128/has miR-21-5p/PTX3, SNHG16, LIN01128, RP11-834C11.4 (LINC02381)/hsa-let-7f-5p/IL6, and LINC00665/hsa-miR-155-5p/PLS1, could be used as potential targets and biomarkers of SSc-ILD. In this study, the role of ceRNA networks in plasma cirexos in SSc was analyzed. The lipid membrane structure of cirexos can prevent degradation of the contents. In cirexos, miRNA, mRNA, and lncRNA have higher concentrations than inside the cells, which is advantageous for diagnosing diseases (13). The main role of lipid molecules in EXOs is to maintain their external morphology, and they can also participate in the biological process of disease development as signal molecules. Several studies have demonstrated the importance of EXOs for the onset or development of SSc. Nakamura et al. (40) found that EXOs were increased in the skin fibroblasts of SSc compared with normal fibroblasts, which may induce the increased expression of type I collagen in the fibroblasts of patients with SSc. In addition, they found that EXOs isolated from the culture medium of fibroblasts from patients with SSc were able to stimulate the expression of type I collagen in normal fibroblasts. Neutrophil-derived EXOs contain many miRNAs and lncRNAs involved in the pathogenesis of SSc. Li et al. (41) found that miRNAs, lncRNAs, and mRNAs in neutrophil-derived EXOs associated with diffuse cutaneous systemic sclerosis (dcSSc) could promote fibrosis by activating the Wnt, AMPK, IL23, and NOTCH signaling pathways. This study not only provides effective biomarkers for the diagnosis of SSc, but also provides potential targets for the mechanism research and clinical treatment of SSc.

Previous studies have found that abnormal accumulation of ECM components can destroy physiological structures leading to organ fibrosis (42, 43). An important process in the development of fibrosis is the transformation of fibroblasts into myofibroblasts, which is driven by many pro-fibrotic factors (44). The activated myofibroblasts can synthesize collagen, resulting in the deposition of ECM collagen and leading to fibrosis (34). Studies have shown that the expression of focal adhesion molecules in the dermis may be important players in SSc pathophysiology (45). Cell adhesion molecules mainly mediate the bidirectional signal transduction between cells and the ECM (45, 46). The key role of cirexos in cell-to-cell communication is mainly related to their cargo, including proteins; these have the ability to activate signaling pathways, which in turn regulate host cell activity and behavior (47). In this study, we found that DEmRNAs in plasma cirexos were mainly enriched in ECM receptor interaction, focal adhesion, and platelet activation pathways. RT-qPCR showed that the hub gene *COL1A1* was upregulated in SSc plasma cirexos. Increased collagen deposition in the alveolar wall leads to progressive destruction of the normal alveolar structure, resulting in increased strength of the ECM (48). In addition, *COL1A1* can induce the differentiation of mesenchymal cells into myofibroblasts through different pathways, which is characterized by the increased expression of α-smooth muscle actin (49). Therefore, *COL1A1* plays an important role in pulmonary fibrosis, and interstitial lung disease (ILD) is an important complication of SSc. Indeed, Velazquez-Enriquez et al. (47) showed that the expression of *COL1A1* in fibroblast-derived exosomes was upregulated in idiopathic pulmonary fibrosis and correlated with the progression of idiopathic pulmonary fibrosis. However, there is a lack of relevant studies on the role of *COL1A1* carried by cirexos in the development of SSc. Based on bioinformatics analysis, we further explored the significance of cirexos in clinical diagnosis. We speculated that the upregulation of *COL1A1* in SSc plasma cirexos may be related to the expression of *COL1A1* in SSc fibroblast-derived exosomes or in the airway ECM of SSc. The expression of *COL1A1* in SSc plasma cirexos may not only be used to diagnose SSc, but may also participate in the biological process of SSc pathogenesis.

Pulmonary complications of SSc, including ILD and pulmonary hypertension (PAH), are the main causes of morbidity and mortality of SSc (50). In this case, there is a great need for biomarkers for diagnosis and prognosis to help clinicians predict the development of SSc and SSc-ILD and provide appropriate treatment for patients. Distler et al. (51) confirmed that high-resolution computed tomography (HRCT) is helpful for predicting the disease progression of SSc-ILD. Therefore, the assessment of the HRCT score is crucial for the diagnosis of fibrosis in SSc. The higher the HRCT score, the more severe the fibrosis. This study found that ENST00000313807 and *COL1A1* in plasma cirexos were positively correlated with the HRCT score, while other RNAs were not statistically correlated with the HRCT score. The expression of HRCT-related genes may represent the degree of lung injury. Christmann et al. (52) confirmed *via* immunohistochemical tests of lung tissue that the expression of *COL1A1* is increased in patients with SSc-ILD, and the high level of type I collagen is positively correlated with the deterioration of the HRCT score. In addition, studies have confirmed that cirexos in patients with SSc can stimulate the expression of genes encoding extracellular matrix components, such as *COL1A1, COL3A1*, and fibronectin-1 (9). Therefore, the expression of ENST00000313807 and *COL1A1* in plasma cirexos may become an important indicator for the evaluation of pulmonary fibrosis, as well as a biomarker for the diagnosis of SSc-ILD.

Activated B cells in SSc promote endothelial cells and fibroblasts to secrete proinflammatory and fibrogenic factors, leading to vascular injury and fibrosis (53). Serum IgM is a soluble marker of B cell activation and one of the diagnostic markers of SSc (53). In this study, we found that ENST00000313807 and hsa-miR-29b-3p in the plasma cirexos of patients with SSc were positively correlated with IgM. Based on our results, we speculated that ENST00000313807 and hsa-miR-29b-3p may be diagnosis markers for the expression of serum IgM in SSc. To date, the most commonly used diagnostic biomarker for SSc is serum autoantibodies. More than 90% of patients with SSc have anti-nuclear antibodies (ANA) in their serum (50). Anti-topoisomerase I (anti-SCL-70) and anti-centromere (anti-CENP-B) antibodies are also highly specific for SSc and SSC-ILD (50). In addition, anti-RO-52 and anti-CENP-A have potential value in the diagnosis of SSc-ILD (54). We found that in plasma cirexos, the expression of ENST00000313807 was positively correlated with Scl-70 and Ro-52, the expression of hsa-miR-29a-3p and hsa-miR-29c-3p was negatively correlated with CENP-B, and *COL1A1* was positively correlated with Ro-52 and CENP-B. Corallo et al. (55) showed that SSc-specific autoantibodies, including anti-Scl-70 and anti-CENP-B, directly induced the increased expression of *COL1A1* in human dermal fibroblasts to promote fibrosis. In addition, our results showed that there is an interaction between ENST00000313807 and hsa-miR-29a-3p, and *COL1A1* and hsa-miR-29a-3p in plasma cirexos. According to our study, we speculated that the ENST00000313807-hsa-miR-29a-3p-*COL1A1* network in SSc plasma cirexos may interact with the high expression of Scl-70, Ro-52, CENP-B, and other specific markers in the serum of patients with SSc, thus affecting the development of SSc fibrosis. The expression of ENST00000313807-hsa-miR-29a-3p- *COL1A1* in SSc plasma cirexos can also be used as a diagnosis marker for the expression of Scl-70, Ro-52, CENP-B, and other specific markers in the serum of patients with SSc. However, few studies have investigated the effect of autoantibodies on pulmonary fibrosis, and further in-depth studies are needed.

Several studies have highlighted that CRP is significantly increased in patients with SSc-ILD. Thus, CRP could be used as an independent biomarker associated with SSc and the presence and severity of ILD (56–58). In this study, we found that the expression of ENST00000313807 was upregulated in SSc plasma cirexos. In SSc plasma cirexos, ENST00000313807 was positively correlated with CRP in SSc serum. Therefore, we speculated that ENST00000313807 in SSc plasma cirexos may be a marker of high CRP expression in SSc serum. Scleroderma renal crisis is a serious and potentially life-threatening complication of scleroderma (1). Urea is an indicator used to evaluate renal function (56). We found that ENST00000313807 in SSc plasma cirexos was positively correlated with urea in SSc serum. Based on the above results, we speculated that the expression of ENST00000313807 in SSc plasma cirexos may be a diagnosis marker of renal function in SSc serum. Both T and B cells participate in abnormal activation of the immune system, which are key factors leading to vascular abnormalities and fibrosis in SSc (59, 60). In addition, several studies have highlighted that the number of lymphocytes is decreased in patients with SSc during the early stage; this is mainly due to scleroderma itself rather than immunomodulation therapy (61–63). Therefore, based on our results, the high expression of ENST00000313807, NON-HSAT194388.1, and *COL1A1*, as well as the low expression of hsa-miR-29a-3p, in SSc plasma cirexos may lead to a lower lymphocyte count in SSc serum and further promote the vascular abnormalities and fibrosis in SSc. The ENST00000313807-hsa-miR-29a-3p-*COL1A1* network in plasma cirexos can also be used as a monitoring index for the expression of peripheral blood lymphocyte subsets in patients with SSc.

Neutrophil infiltration in diseased tissues is an important factor in fibrosis. In chronic inflammatory or autoimmune diseases, the high expression of inflammatory cytokines drives neutrophils to form neutrophil extracellular traps (NETs). The number of neutrophils in peripheral blood may reflect the infiltration of neutrophils into tissues (64). Our results showed that ENST00000313807 and NON-HSAT194388.1 in plasma cirexos were positively correlated with the absolute value and percentage of neutrophils in serum. However, hsa-miR-29a-3p in the plasma cirexos was negatively correlated with the percentage of neutrophils in serum. Tu et al. (64) found that the number of neutrophils in peripheral blood of patients with SSc increased significantly. Wareing et al. (65) observed that higher neutrophil counts predicted a worse ILD course and higher long-term mortality in patients with SSc-ILD. In addition, Chikhoune et al. (56) showed that the absolute neutrophil count was significantly increased in patients with dcSSc or ILD. Kase et al. (66) used fractional analysis of bronchoalveolar lavage (FBAL) to analyze the bronchoalveolar lavage fluid of patients with SSc-ILD. The results showed that there were more neutrophils in the FBAL-3 of SSc-ILD patients with anti-SCL-70 autoantibodies than those without anti-SCL-70 autoantibodies. A higher percentage of neutrophils in FBAL-3 is associated with the development of end-stage SSc-ILD. Cakmak et al. (67) performed FBAL, HRCT, pulmonary function tests, and dyspnea measurements in 65 patients with progressive SSc. The results suggest a correlation between NEUT% and honeycombing of the lung. Therefore, NEUT% is associated with fibrosis in SSc. In this study, ENST00000313807 and NON-SAT194388.1 were found to be positively correlated with NEUT%, and hsa-miR-29a-3p was found to be negatively correlated with NEUT%. Therefore, the results of the present study indicate that ENST00000313807, NON-HSAT194388.1, and hsa-miR-29a-3p in plasma cirexos could also be used as monitoring indicators of serum neutrophil content in regard to the occurrence and development of SSc.

The red cell distribution width (RDW) is a biomarker to quantify the abnormal size of red blood cells in peripheral blood and has value as a biomarker to assess the severity of vascular damage (68). We found that the expression of hsa-miR-29a-3p in plasma cirexos was negatively correlated with the standard deviation of RDW in serum. Farkas et al. (68) found that increased RDW was associated with dcSSc. Therefore, hsa-miR-29a-3p may have an influence on RDW in SSc, which is an indicator of the vascular damage in SSc. In this study, hsa-miR-29a-3p was found to be negatively correlated with RDW-SD, while there was a lack of references regarding RDW-SD expression in patients with SSc. Patients with SSc often suffers from gastrointestinal tract damage, characterized by atrophy of smooth muscle and decreased intestinal motility, which is mainly caused by autonomic nervous dysfunction. These changes significantly affect intestinal transport and nutrient absorption, resulting in malnutrition caused by malabsorption (69). Serum albumin, hemoglobin, and body mass index reflect nutritional status. Paolino et al. (69) found that the expression of serum hemoglobin and albumin was significantly reduced in malnourished patients with SSc. In addition, Chikhoune et al. (56) found that hypoalbuminemia was related to the skin and lung severity of SSc. However, few studies have investigated the expression of the albumin/globulin ratio in patients with SSc. Therefore, according to the results of this study, we speculated that the high expression of ENST00000313807, NON-HSAT194388.1, and *COL1A1* in plasma cirexos of patients with SSc, and the low expression of I-miR-29a-3p and has-miR-29b-3p may be indices for the expression of serum albumin and albumin/white in patients with SSc.

In fibroblasts derived from scar skin and skin fibroblasts induced by lipopolysaccharides, IL-10 downregulated the expression of toll-like receptor 4 [TLR4hosphorpho-NF-κB p65 (pp65) collagen type I, collagen type III, and α-smooth muscle actin (70)]. Therefore, IL-10 can regulate the TLR4/NF-κB pathway in dermal fibroblasts through the IL-10 receptor (IL-10R)/STAT3 axis to reduce both ECM protein deposition and fibroblast transformation to myofibroblasts, thereby inhibiting the formation of skin scar induced by lipopolysaccharide. In addition, Thoreau et al. (71) found that the expression of IL-10 was reduced in mouse models and patients with SSc and was associated with ILD formation. We found that the expression of *COL1A1* in SSc plasma cirexos was negatively correlated with IL-10 in the SSc serum, which is consistent with the abovementioned studies. However, the mechanism needs to be further verified.

miR-29a inhibits the expression of collagen in human fetal scleral fibroblasts by regulating the Hsp47/Smad3 signaling pathway (72). Jafarinejad-Farsangi et al. (73) found that the expression of has-miR-29a was downregulated in dermal fibroblasts of dcSSc patients and dermal fibroblasts treated with transforming growth factor (TGF)-β *in vitro*. In addition, hsa-miR-29a was able to effectively reduce TGF-β-induced collagen production in dermal fibroblasts. Therefore, hsa-miR-29a may serve as a therapeutic target for SSc and other fibrotic diseases with abnormal collagen expression. In this study, hsa-miR-29a-3p was downregulated and COL1A1 was upregulated in patients with SSc. hsa-miR-29a-3p-COL1A1 can be used as a therapeutic target for SSc.

Increasing studies have investigated non-invasive biomarkers to diagnose, evaluate, and treat SSc; however, few of these biomarkers are used clinically (8). Compared to circulating non-coding RNAs in the serum or plasma, the abnormal expression of non-coding RNAs carried by cirexos in SSc have the advantages of stability and practicability as novel biomarkers and therapeutic targets for the diagnosis and treatment of SSc (13, 74, 75).

## 5. Conclusion

In this study, the ROC curve results suggested that the combined diagnosis of the four lncRNA–miRNA–mRNA networks of plasma cirexos had more advantages than the independent diagnosis of SSc. The ENST00000313807-hsa-miR-29a-3p-*COL1A1* network was correlated with the clinical data of patients with SSc. These indicators play an important role as biomarkers in the diagnosis of SSc. The ENST00000313807-hsa-miR-29a-3p-*COL1A1* network exhibited the most potential as a combined diagnosis biomarker for the clinical diagnosis and treatment of SSc. It is worth noting that our results showed that ENST00000313807 interacts with hsa-miR-29a-3p, which interacts with *COL1A1*, although the mechanism still needs to be uncovered.

## Data availability statement

The data presented in the study are deposited in the Gene Expression Omnibus (GEO) repository, accession number GSE224884.

## Ethics statement

The studies involving human participants were reviewed and approved by the Ethics Committee of the First Affiliated Hospital of Baotou Medical College, Inner Mongolia University of Science and Technology [Approval No. 2018 (017)]. The patients/participants provided their written informed consent to participate in this study.

## Author contributions

XS and TD designed and performed the experiments. XS, TD, BW, and HF analyzed the data. XS, TD, ZC, LG, and YW reviewed and edited the manuscript. All authors have contributed to read and approved the submitted version.

## Footnotes

1
https://www.genecards.org/

2
https://www.disgenet.org/home/

3
https://bioinfogp.cnb.csic.es/tools/venny/index.html

4
https://www.gsea-msigdb.org/gsea/index.jsp

5
https://cytoscape.org/

6
http://bioinfo.life.hust.edu.cn/lncRNASNP

7
https://starbase.sysu.edu.cn/

8
https://string-db.org/

9
http://ctdbase.org/

10
http://www.csbio.sjtu.edu.cn/bioinf/lncLocator/

11
http://bio-bigdata.cn/mRNALocater/

12
http://s.tartaglialab.com/page/catrapid_group

13
https://pubmed.ncbi.nlm.nih.gov/
